# Ultrasound and Sialendoscopy Findings in Radioactive Iodine–Induced Sialadenitis: Comparative Analysis and Possible Impact on Management

**DOI:** 10.3390/jcm13030657

**Published:** 2024-01-23

**Authors:** Michael Koch, Sarina Müller, Konstantinos Mantsopoulos, Heinrich Iro, Matti Sievert

**Affiliations:** Department of Otorhinolaryngology and Head and Neck Surgery, University of Erlangen–Nuremberg, 91054 Erlangen, Germany; sarina.mueller@uk-erlangen.de (S.M.); konstantinos.mantsopoulos@uk-erlangen.de (K.M.); heinrich.iro@uk-erlangen.de (H.I.); matti.sievert@uk-erlangen.de (M.S.)

**Keywords:** ultrasound: sialendoscopy, salivary gland, radioactive iodine, treatment

## Abstract

Background: To assess the correlation/association between ultrasound and sialendoscopy findings in radioactive iodine therapy–induced sialadenitis (RAIS). Methods: Patients presenting with RAIS were investigated with ultrasound and sialendoscopy. Four pathologic ultrasound parameters and seven pathologic sialendoscopy parameters were retrospectively assessed. Correlations/associations between ultrasound and sialendoscopy findings and associations between the changes between the first and last ultrasound and sialendoscopy findings were assessed separately for the parotid (PG) and submandibular glands (SMG). Results: Sixty-seven patients were included. In the first examination, 107 glands were investigated (PGs 88.8%, SMGs 11.21%), and in the last examination, 64 glands were investigated (90.6% PGs, 9.4% SMGs). Highly significant positive associations were observed between the severity or category of ultrasound and sialendoscopy findings for first and last examinations for PGs (both *p* = 0.0001) and SMGs (*p* = 0.002; *p* = 0.037). Duct dilation had a significant negative association with the sialendoscopy findings for PGs in the first and last examinations (both *p* = 0.0001), but not for SMGs. Comparison of changes in the ultrasound and sialendoscopy findings between the first and last examinations showed a significant positive association for PGs (*p* = 0.0001) but not for SMGs. Conclusions: Ultrasound and sialendoscopy findings for the parenchyma and duct system in RAIS showed significant associations/correlations and can be useful for effective management in RAIS.

## 1. Introduction

Radioactive iodine–induced sialadenitis (RAIS) in the major salivary glands is observed in 10–60% of patients after radioactive iodine treatment for differentiated thyroid cancer [1,2,3,4,5,6,7,8,9,10,11,12,13,14,15,16]. RAIS is caused by the uptake of I^131^ into the salivary glands via the Na^+^/K^+^/2Cl^–^-symporter system, which allows active ATP-dependent cumulation. Damage to acinic cells is caused primarily by I^131^ but also secondarily by duct obstruction due to strictures and stenoses [2,10,17,18,19,20,21]. The parotid glands (PGs) are affected unilaterally in 75–90% of cases and bilaterally in 25%. The submandibular glands (SMGs) are affected in less than 50% of cases [4,10,11,15,21,22].

Ultrasonography (US) and sialendoscopy (SE) play an important role in the management of RAIS. Various ultrasound parameters for diagnosing RAIS have been described (hypoechoic/heterogeneous/atrophic parenchyma, duct dilation), and the findings after radioactive iodine treatment (RAIT) are time-dependent and dose-dependent [5,23,24,25,26,27,28]. This was particularly shown in a recent publication by our study group [29].

Sialendoscopy is reported to be a valuable diagnostic and therapeutic tool in RAIS. Relevant findings include paleness of the mucosa, duct inflammation, debris, plaques, stricture/stenosis, and duct obstruction [21,22,30,31,32,33,34,35,36,37,38]. Ultrasound has only been included in a few of these publications, without more detailed analysis [22,32,35].

To the best of our knowledge, no publications to date have investigated whether and in what ways the ultrasound and sialendoscopy findings correlate in RAIS. In one report, findings were based on sialographic and sialendoscopic findings, classified into three groups (mild 12.31%, moderate 35.4%, and severe 52.3%) [38].

The aims of the present study were to classify ultrasound and sialendoscopy findings in RAIS and investigate possible correlations/associations between them in order to assess the severity and prognosis of RAIS, as this may be helpful in managing the condition.

## 2. Methods

This retrospective study was carried out at the Department of Otorhinolaryngology and Head and Neck Surgery at the Friedrich-Alexander-University of Erlangen–Nuremberg. This study was conducted according to the guidelines of the Declaration of Helsinki and approved by the Institutional Review Board Approval of the Friedrich-Alexander-University of Erlangen–Nuremberg. Informed consent was obtained from all subjects involved in this study. The database was searched for patients who were examined with ultrasound and sialendoscopy in the department’s ultrasound unit after RAIT for differentiated thyroid cancer.

The inclusion criteria were: (1) for patients: adequate follow-up, complete data available; no treatment/status post-radiotherapy/chemotherapy for other malignancies in the head and neck region; and (2) for glands: no treatment for inflammatory and/or obstructive salivary diseases; no prior surgery or other surgical manipulations in the salivary glands.

The clinical parameters included were: age, gender, type, and number of major salivary glands/cases involved; time after RAIT at first presentation (months); and cumulative therapeutic RAIT dose (MBq).

**Figure 1:**
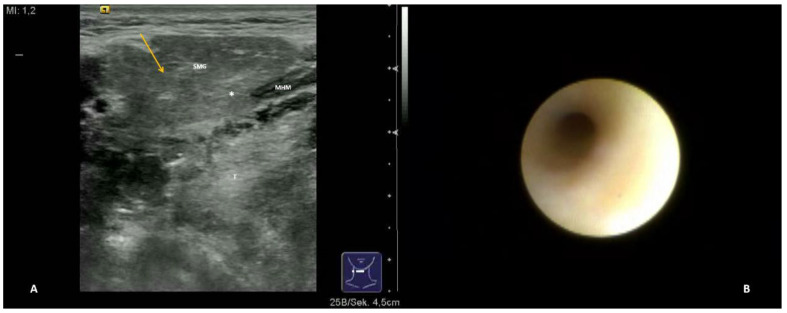
US (**A**) shows right SMG with slight hypoechoic changes (arrow) with normal echotexture in a small part (white asterix). The corresponding SE (**B**) depicts chronic SD: the duct wall appears pale with no narrowing of the lumen (Supplemental Video S1). SMG, submandibular gland; MHM, mylohyoid muscle; T, tonsil.

**Figure 2:**
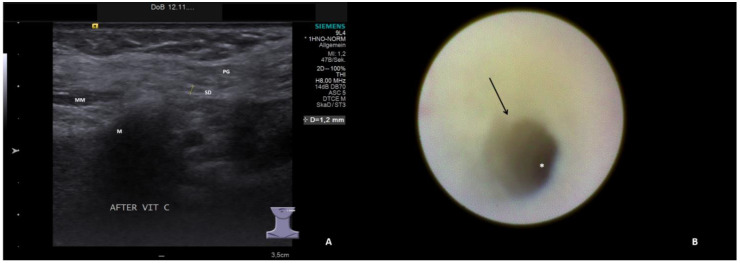
US (**A**) shows left PG with heterogeneous parenchyma and duct dilation (1.2 mm, dotted line) after stimulation. The corresponding SE (**B**) depicts chronic SD with tendency to stenosis: the duct wall appears pale with narrowing (arrow) of the lumen (asterix, Supplemental Video S2). PG, parotid gland; SD, Stensen’s duct; M, mandible; MM, masseter muscle.

**Figure 3:**
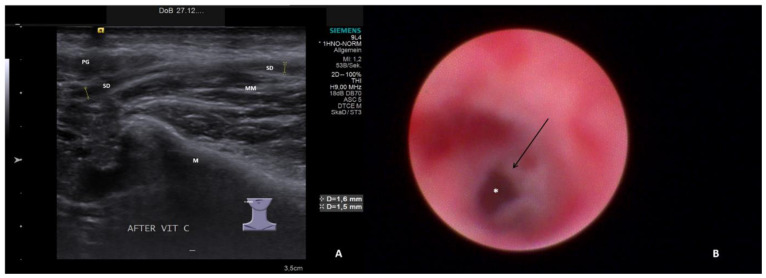
US (**A**) shows right PG with heterogeneous parenchyma and duct dilation (1.5 and 1.6 mm, dotted lines) after stimulation. The corresponding SE (**B**) depicts subacute SD with more fibrotic than inflammatory stenosis (arrow) with narrowing of the lumen (white asterix, Supplemental Video S3). PG, parotid gland; SD, Stensen’s duct; M, mandible; MM, masseter muscle.

**Figure 4:**
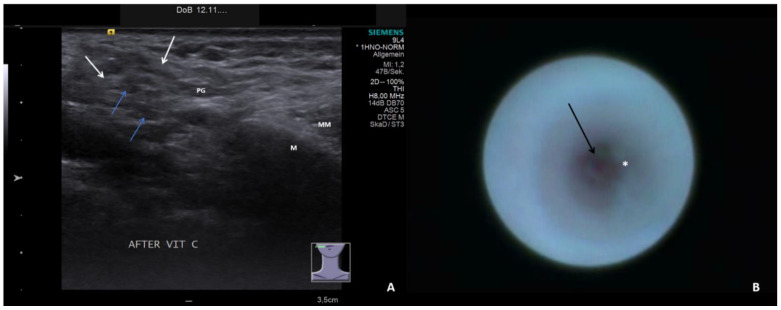
US (**A**) shows right PG with heterogeneous parenchyma with residual parenchyma (white arrows) and fatty degeneration (blue arrows) and without duct dilation after stimulation. The corresponding SE (**B**) depicts a chronic SD with a diffusely thickened wall (white asterix) leading to fibrotic stenosis (arrow, Supplemental Video S4). PG, parotid gland; M, mandible; MM, masseter muscle.

**Figure 5:**
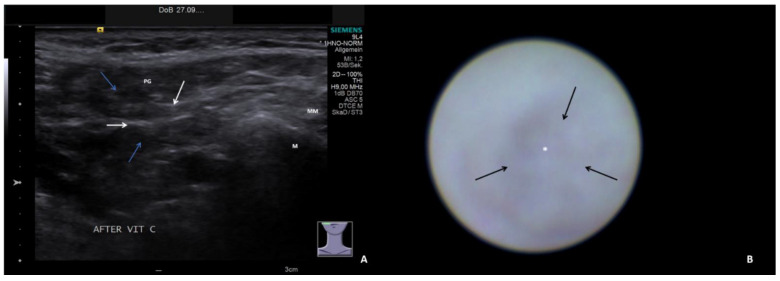
US (**A**) shows right PG with atrophic parenchyma with mixed hyper-echoic (fibrosis, white arrows) and anechoic parenchyma (fatty degeneration, blue arrows) and no duct dilation after stimulation. The corresponding SE (**B**) depicts a complete fibrotic stenosis without any duct lumen visible (asterix; black arrows indicate the area of stenosis; the Supplemental Video S5 shows duct obliteration after opening of the stenosis). PG, parotid gland; M, mandible; MM, masseter muscle.

The ultrasound examinations were conducted or supervised by certified otolaryngologists using high-end ultrasound devices (Sonoline Elegra until 2011, ACUSON S2000/S3000 from 2012 to 2019, ACUSON Sequoia from 2019 to 2022; Siemens Medical Solutions USA Inc., Malvern, PA, USA) and linear transducers at 4–9 MHz (9L4) or 4–10 MHz (10L4). Ultrasound parameters were classified into normal findings (slightly hyperechoic, no inflammatory changes) and four categories relative to increasing pathologic signs for parenchyma and ducts, as described in one recent publication [29]:

1. Hypoechoic and homogeneous (no relevant loss of gland parenchyma; Figure 1A)
2. Heterogeneous/coarse (partial loss of parenchyma with residual parenchymal echotexture recognizable; Figure 2A, Figure 3A and Figure 4A)
3. Mixed hypoechoic/hyperechoic, without relevant parenchymal tissue visible (replacement of gland parenchyma by fatty-fibrotic tissue, parenchymal atrophy, Figure 4A and Figure 5A).
4. The presence of duct dilation (≥1–2 mm), with or without gland stimulation, was noted as a sign of (residual) gland function (Figure 1A, Figure 2A, Figure 3A, Figure 4A and Figure 5A).

Sialendoscopy was performed using various semirigid sialendoscopes (types: 11572, 0.8 mm; 11574, 1.1 mm; Tuttlingen, Germany) [39,40,41]. Sialendoscopy findings were classified into normal findings and seven categories for increasing pathologic signs. All relevant findings reported in the literature (paleness of the mucosa, inflammation, debris, plaques, stricture/stenosis, severe stenosis, total obstruction) [21,22,30,31,32,33,34,35,36,37,38] were included. The categories for pathologic findings were:

1. Chronic sialodochitis (SD: inflammation, paleness of the duct wall, plaques, discharge; Figure 1B; Supplementary Video S1)
2. Sialodochitis with a tendency toward duct narrowing (SD + Sten: SD and thickness of the duct wall with a tendency toward luminal narrowing; Figure 2B; Supplementary Video S2)
3. Short duct stenosis (≤1 cm; Figure 3B; Supplementary Video S3)
4. Segmental duct stenosis (≥1 cm)
5. Long duct stenosis (≥3 cm; Figure 4B; Supplementary Video S4)
6. Complete duct stenosis (no lumen visible, but visible lumen proximal to the stenosis)
7. Duct obliteration (lumen obstructed by fibrotic, cobweb-like material, no epithelialized lumen visible; Figure 5B; Supplementary Video S5) as a sign of gland atrophy (diffuse long stenosis and/or complete duct stenosis with proximal duct obliteration)

**Figure 6:**
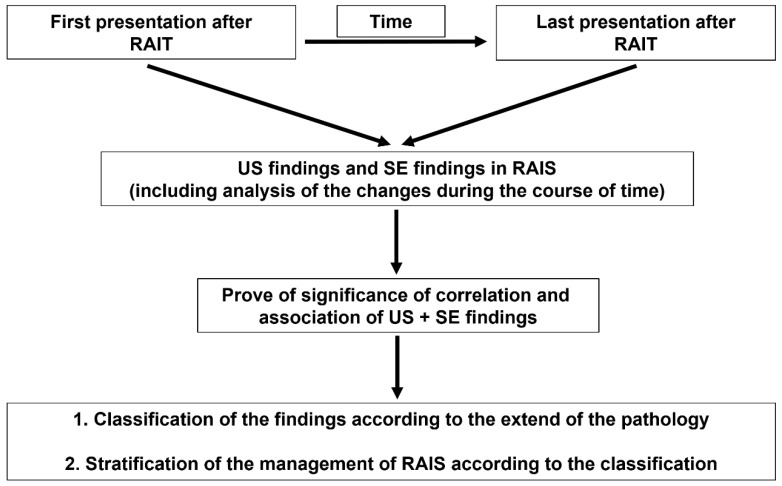
Flow Diagram of the Study.

Relevant ultrasound and sialendoscopy findings were stored on video for later assessment. If several examinations were performed, only the first and last were included. The maximum ultrasound and sialendoscopy findings were noted and compared. The time in months from the end of the RAIT to the first ultrasound and sialendoscopy examinations and between the first and last ultrasound and sialendoscopy examinations was noted. If identical glands were investigated at the first and last presentations, changes in the ultrasound and sialendoscopy findings were noted (improved, equivalent, or worse) and compared using correlation and association tests (Figure 6).

### Statistical Analysis

IBM SPSS Statistics for Windows, version 26, was used (IBM Corporation, Armonk, NY, USA). All data are given as the mean plus or minus the standard error of the mean, median, and range. Bivariate correlations were calculated using Pearson’s correlation coefficient. Differences/associations between the groups for categorical variables were calculated using the chi-square exact test. The significance level was set at *p* ≤ 0.05.

## 3. Results

Sixty-seven patients for whom all relevant data were available were included. Their mean age was 50.1 ± 1.46 years (median 49.0, range 22–83); 89.6% were women (60/67). All of the patients presented because of one or several symptoms starting after a mean of 10.5 ± 2.99 months (median 5, range 1–144) after RAIT. The mean therapeutic dose applied was 7793.1 ± 631.6 MBq (median 5500.0, range 3000–25,796). The first presentation for ultrasound and sialendoscopy after RAIT was after a mean of 27 ± 3.9 months (median 13, range 1.5–188) in PGs and 73.1 ± 26.1 months (median 28, range 4–254) in SMGs. PGs were investigated in 57 cases (85.1%) and SMGs in seven cases (10.4%), and both glands were examined in the same patient in three cases (4.5%). Bilateral glands were investigated in 37 cases (55.2%).

A total of 171 glands were examined using ultrasound and sialendoscopy (153 PGs, 89.5%; 18 SMGs, 10.5%). This included first examinations (107 glands: 95 PGs, 88.8%; 12 SMGs, 11.2%; bilateral 35 of 57 PG cases, 61.4%; and two of 10 SMG cases, 20%) and last examinations (64 glands: 58 PGs, 90.6%; six SMGs, 9.4%; bilateral 14 of 44 PG cases, 31.8%).

### 3.1. Ultrasound and Sialendoscopy Findings and Correlations/Associations

Normal tissue on ultrasonography was observed only in PGs (first US 10.8%; last US 13.8%). The frequency of hypoechoic parenchyma (40% vs. 16.8%) and heterogeneous parenchyma (32.6% vs. 27.6%) decreased between the first and last examinations, but atrophic echotextures increased (16.8% vs. 43.1%). For SMGs, normal findings were not observed. Atrophy was not observed at the first ultrasound examination, but in the last one in 33.3% (Table 1).

No evident pathologic changes were observed at the first sialendoscopy in only one PG and in none of the glands at the last sialendoscopy. SD + Sten was observed in both glands in 30–40% of cases at the first sialendoscopy and in 20–33% of cases at the last. Stenoses (all kinds) were the pathological condition most often observed at the first sialendoscopy and the second most often at the last sialendoscopy in both glands (PG: 41.1% and 22.4%; SMG: 50% and 33.3%), with short or long stenoses being most frequent. Duct obliteration was the single pathology most often observed in PGs at the first and last sialendoscopy (25.3% and 53.4%), but was only observed in SMGs at the last sialendoscopy in one case (Table 1).

There were significant correlations between increasing categories of ultrasound and sialendoscopy findings for the first, last, and first and last examinations combined for PGs (first: 0.77, *p* = 0.0001; last: 0.91, *p* = 0.0001; first and last: 0.84, *p* = 0.0001). For SMGs, this type of correlation was only observed at the last examination and when both examinations were analyzed (last: 0.84, *p* = 0.038; first and last: 0.67, *p* = 0.002).

Normal parenchyma was not observed in SMGs and was only seen at the first ultrasound examination in PGs. It was associated with a sialendoscopy-based diagnosis of sialodochitis in 90% of cases. No typical pathologic changes in the visible duct system were also noted in one case. Hypoechoic parenchyma in both glands at the first and last ultrasound examinations was associated with lower categories of sialendoscopy findings (any kind of stenosis, 92.3%, or SD + Sten, 100%). Heterogeneous parenchyma in PGs was associated both with lower categories (first SE 45.83%; last SE 62.5%) and also with duct obliteration (first SE 54.17%; last SE 37.5%). Atrophy of the parenchyma was strongly associated with duct obliteration (81.25% at first SE; 100% at last SE). In PGs, duct obliteration was the single pathology most often observed at the first sialendoscopy (25.3%) and was associated with mixed parenchymal changes (45.8% heterogeneous and 54.2% atrophic). Its frequency increased to 53.4% at the last sialendoscopy, with a higher percentage of atrophic parenchyma (80.6%) in comparison with heterogeneous parenchyma (19.4%, Table 2).

By contrast, heterogeneous parenchyma at the first sialendoscopy in SMGs was associated only with lower categories of sialendoscopic findings (short stenosis within the hilum in 50%, associated with hypoechoic and heterogeneous parenchyma), and atrophy was not observed. Atrophy was observed at the last sialendoscopy in two cases (complete stenosis and duct obliteration, 50% each, Table 2). Taken together, the ultrasound categories showed a significant positive association with the sialendoscopy categories for PGs (first examinations, *p* = 0.0001; last examinations, *p* = 0.0001), but not for SMGs. Only weak significance was noted here when the findings for the first and last ultrasound and sialendoscopy examinations were summarized (*p* = 0.037).

### 3.2. Duct Dilation and Maximum Ultrasound Findings

Duct dilation was present at the first examination in 61.68% of cases, with the highest frequency if the parenchyma was hypoechoic (82.22%) or heterogeneous (66.67%), but lowest in atrophic parenchyma (6.25%). Frequencies were higher in PGs in comparison with SMGs. At the last examination, the frequency was 36.2% for PGs, again with hypoechoic and heterogeneous parenchyma showing the highest frequencies (66.67% and 62.5%). No duct dilation was observable in SMGs (Table 3). A significant association between the category of the ultrasound findings and the presence of duct dilation was noted for PGs (first US: *p* = 0.0001; last: *p* = 0.003), but not for SMGs.

### 3.3. Duct Dilation and Maximum Sialendoscopy Findings

Duct dilation was highest at the first examination if SD + Sten (80% PGs, 33.33% SMGs), short stenoses (95% PGs, 33.3% SMGs), or long stenoses (80% PGs) were present. It was observed in 20.83% of glands showing duct obliteration. At the last examination, duct dilation was observed only in PGs, particularly if SD + Sten (83.33%), short stenoses (83.33%), or long stenoses (57.14%) were present (Table 4). A significant association between the category of the sialendoscopy findings and the presence of duct dilation was noted for PGs (first US: *p* = 0.0001; last: *p* = 0.003), but not for SMGs.

### 3.4. Changes between First and Last Ultrasound and Sialendoscopy

The last examination was performed in PGs after a mean of 31.8 ± 5.5 months (median 15.5, range 2–177) and in SMGs after a mean of 49.5 ± 20.7 months (median 24.5, range 8–115) after the first examination. In comparison with the first examination, the ultrasound findings in 58 PGs deteriorated in 48.3% of cases (28/58), were unchanged in 43.1% (25/58), and improved in 8.6% (5/58). In six SMGs, 33.3% (2/6) deteriorated and 66.7% (4/6) were unchanged (for details, see Table 5). Altogether, changes in the categories of ultrasound and sialendoscopy findings between the first and last examinations showed a highly significant positive association for PGs (*p* = 0.0001) but not for SMGs (*p* = 0.300). Hypoechoic parenchyma (40% first US, 15.5% last), duct dilation (64.2% first US, 36.2% last), and lower categories of sialendoscopy findings (SD + Sten, all kinds of stenoses: 73.7% first sialendoscopy, 46.6% last) were all present with higher frequencies at the first examination in comparison with the last. By contrast, atrophic parenchyma (16.8% first US, 41.4% last) and duct obliteration (25.3% first SE, 53.4% last) were observed more often at the last examination in comparison with the first (for details, see Table 5).

## 4. Discussion

Ultrasound [5,23,24,25,26,27,28,29] and sialendoscopy [21,22,30,31,32,33,34,35,36,37,38] are among the most important tools for managing RAIS. Both the parenchyma and the duct system can be assessed using ultrasound and sialendoscopy. Hypoechoic parenchyma [5,23,24,25,26,28], heterogeneous parenchyma [5,24,25,26,28], parenchymal loss (gland atrophy) [5,23,24,26], and duct dilation [5] have been described using ultrasound [29]. Sialendoscopy findings reported have included paleness of the mucosa, duct inflammation, debris, plaques, stricture/stenosis, severe stenosis, and total duct obliteration [21,22,30,31,32,33,34,35,36,37,38].

Ultrasound has been used as a diagnostic tool in RAIS investigated by sialendoscopy [22,32,35], but to the best of our knowledge, there have been no studies categorizing and comparing ultrasound and sialendoscopy findings. On the basis of sialography and sialendoscopy, Li et al. classified findings in RAIS into three groups: mild (stenosis and ectasia in the main duct, 0.9 mm sialendoscope passing easily, present in 12.3%); moderate inflammation (one point of severe stenosis in the main duct, where the 0.9 mm sialendoscope could not pass, present in 35.4%); and severe inflammation (two points or more of severe stricture or diffuse strictures in the main duct, present in 52.3%) [38]. However, this classification did not include findings for the gland parenchyma. As RAIS is caused by increased uptake of I^131^ into acinic and ductal cells instead of Cl^–^ due to the Na^+^/K^+^/2Cl^–^-cotransporter, both parenchymal and duct cells are damaged [2,17,19]. Consequently, a classification should include ductal and parenchymal findings.

The present study shows that there are significant correlations and associations between ultrasound and sialendoscopy findings after RAIT (Table 1, Table 2, Table 3 and Table 4). On the basis of the current literature, parenchymal/ductal ultrasound findings can be classified into four main pathologic categories [5,23,24,25,26,27,28] and sialendoscopy findings into seven main pathologic categories [21,22,30,31,32,33,34,35,36,37,38].

Highly significant positive correlations between the severity of the ultrasound and sialendoscopy findings were noted for PGs (first, last, and both examinations: *p* = 0.0001 each), but for SMGs the associations were weaker (last: *p* = 0.038, both examinations: *p* = 0.002) (Table 1 and Table 2).

Duct dilation was negatively associated with a higher category of parenchymal ultrasound findings in the PGs at the first (*p* = 0.0001) and last (*p* = 0.003) examinations, but not in the SMGs (no atrophy present). Duct dilation was associated with normal, hypoechoic, and heterogeneous parenchyma in 98.4% of PGs and 100% of SMGs at the first examination and in 80.95% of PGs at the last examination (Table 3).

Duct dilation also showed a significant negative association with the severity of the sialendoscopy findings for PGs at the first examination (*p* = 0.0001) and last examination (*p* = 0.014). No significant differences were noted for SMGs in relation to the ultrasound findings. At the first and last PG examinations, duct dilation was most frequent in SD + Sten (80% and 83.3%), short stenoses (95% and 83.3%), and long stenoses (80% and 57.1%), and in around 20% of cases of duct obliteration. In the SMGs, duct dilation was only observed at the first ultrasound examination (only lower categories of sialendoscopy findings), but in no cases at the last (mixed pathologies; Table 4).

A significant positive association was seen between changes in the various categories of ultrasound and sialendoscopy findings for PGs (*p* = 0.0001) but not for SMGs. In the PGs, the ultrasound and sialendoscopy findings deteriorated in 48.3% and 50% of cases, were unchanged in 43.1% and 37.9%, and improved in 8.6% and 12.1%, respectively. Notably, the observation by Roh et al. that there were no cases of regression of ultrasound changes at the follow-up investigations [25] also applied to almost 90% of cases in the present study—but not all cases (Table 5).

The results presented in this study show that the comprehensive information regarding the state of the parenchyma and duct system that can be obtained using ultrasound and sialendoscopy can be applied in the management of RAIS. This has not been addressed in detail in earlier reports. [21,22,30,31,32,33,34,35,36,37,38]

- Normal parenchyma with unremarkable findings, SD, SD + Sten on sialendoscopy: the prognosis tends to be good and treatment measures are indicated. Treatment consists of conservative measures (gland massage with sialogouges) and (sialendoscopic-controlled) irrigation of the duct system with cortisone.
- Hypoechoic glands and SD, SD + Sten, or any kind of stenosis: a relatively good prognosis can be expected. Treatment measures are worthwhile. In addition to the afore-mentioned measures, interventional sialendoscopy with the opening and dilation of a stenosis with or without stent implantation can be indicated.
- Heterogeneous parenchyma and presence of SD + Sten or any kind of stenosis: the prognosis tends to be uncertain. Treatment may be tried, particularly if duct dilation is present, but RAIS may impair the subsequent course. Conservative measures, irrigation of the duct system, and interventional sialendoscopy may belong to the therapeutic measures.
- Heterogeneous parenchyma and complete or long stenosis, or incipient signs of duct obliteration: progression is more likely. The success of any therapy may not be promising (at least in the long term), particularly if no duct dilation is visible. While conservative measures like irrigation of the duct system may be performed, the benefit of interventional sialendoscopy is questionable.
- Atrophic parenchyma and duct stenosis, or duct obliteration with or without duct dilation: no further therapeutic measures (except conservative) are indicated.

The value of ultrasound and sialendoscopy is also highlighted by the fact that both are cost-effective. They can both be conducted with the patient under local anesthesia and can be repeated as often as needed, as they are not invasive, or at least less invasive. The findings can be stored on video for additional analysis. A recent cost-effectiveness analysis of diagnostic measures in RAIS, including ultrasound and sialendoscopy, found that ultrasound is the least expensive method. It was also the most cost-effective tool for diagnosing RAIS in comparison with magnetic-resonance and computed-tomographic sialography. Initial sialendoscopy was found to be more cost-effective in comparison with medical management using diagnostic ultrasound [42].

The present study has some limitations. Firstly, it is a single-center study and a retrospective analysis of patients presenting with symptoms in the major salivary gland after RAIT at our salivary gland center; there may therefore be some referral bias. The examinations were performed at different times after RAIT, when the patients were referred or presented. Secondly, the first and last ultrasound and sialendoscopy examinations were selected arbitrarily so that the changes expected after increasing follow-up periods could also be assessed [1,2,12,14].

## 5. Conclusions

As noninvasive or less invasive, cost-effective modalities, ultrasound and sialendoscopy provide relevant findings in the parenchyma and duct system in patients with RAIS. The findings can be classified relative to the increasing pathology in RAIS. Using the classification parameters shows that the ultrasound and sialendoscopy findings are significantly associated and/or correlated. Ultrasound and sialendoscopy findings, particularly in combination, may be very useful for the management of RAIS and the assessment of the further prognosis.

## Figures and Tables

**Table 1:** Primary data after first and last ultrasound and sialendoscopy examinations of parotid glands and submandibular glands in 67 patients after radioactive iodine therapy.

Findings	All (n = 107)	PGs (n = 95)	SMGs (n = 12)
Maximum US findings at *first* examination			
Normal parenchyma	10 (9.3%)	10 (10.5%)	0
Hypoechoic parenchyma	45 (42.1%)	38 (40.0%)	7 (58.3%)
Heterogeneous parenchyma	36 (33.6%)	31 (32.6%)	5 (41.7%)
Atrophic parenchyma	16 (15.0%)	16 (16.8%)	0
Duct dilation	66 (61.7%)	61 (64.2%)	5 (41.7%)
Maximum SE findings at *first* examination			
Normal	2 (1.9%)	1 (1.1%)	1 (8.3%)
Sialodochitis	18 (16.8%)	16 (16.8%)	2 (16.7%)
Sialodochitis with tendency to stenosis	18 (16.8%)	15 (15.8%)	3 (25.0%)
Stenosis (short)	26 (24.3%)	20 (21.1%)	6 (50.0%)
Stenosis (segmental)	2 (1.9%)	2 (2.1%)	0
Stenosis (long)	15 (14.0%)	15 (15.8%)	0
Stenosis (complete, without obliteration)	2 (1.9%)	2 (2.1%)	0
Duct obliteration	24 (1.9%)	24 (25.3%)	0
Maximum US findings at *last* examination	All (n = 64)	PGs (n = 58)	SMGs (n = 6)
Normal parenchyma	8 (12.5%)	8 (13.8%)	0
Hypoechoic parenchyma	11 (17.2%)	9 (15.5%)	2 (33.3%)
Heterogeneous parenchyma	19 (29.7%)	16 (27.57%)	2 (33.3%)
Atrophic parenchyma	26 (40.6%)	25 (43.10%)	2 (33.3%)
Duct dilation	21 (32.8%)	21 (36.2%)	0
Maximum SE findings at *last* examination			
Normal	0	0	0
Sialodochitis	8 (13.8%)	8 (13.8%)	0
Sialodochitis with tendency to stenosis	8 (13.8%)	6 (10.3%)	2 (33.3%)
Stenosis (short)	8 (13.8%)	6 (10.3%)	2 (33.3%)
Stenosis (segmental)	0	0	0
Stenosis (long)	7 (10.9%)	7 (12.1%)	0
Stenosis (complete, without obliteration)	1 (1.6%)	0	1 (16.7%)
Duct obliteration	32 (50.0%)	31 (53.4%)	1 (16.7%)

PG, parotid gland; SE, sialendoscopy; SMG, submandibular gland; US, ultrasound.

**Table 2:** Cross-tabulation: findings after first and last ultrasound and sialoendoscopy examinations of parotid glands and submandibular glands in 67 patients after radioactive iodine therapy.

SE Findings	Normal	Sialodochitis	Sialodochitis with Tendency to Stenosis	Stenosis (Short)	Stenosis (Segmental)	Stenosis (Long)	Stenosis (Complete, Without Obliteration)	Duct Obliteration	*Total*
*First* US examination: maximum US findings									
All glands									
Normal parenchyma	1	9	–	–	–	–	–	–	10 (9.3%)
Hypoechoic parenchyma	1	8	14	14	2	5	1	–	45 (42.1%)
Heterogeneous parenchyma	–	1	4	11	–	8	1	11	36 (33.6%)
Atrophic parenchyma	–	–	–	1	–	2	–	13	16 (14.95%)
*Total*	**2 (1.9%)**	**18 (16.8%)**	**18 (16.8%)**	**26 (24.3%)**	**2 (1.9%)**	**15 (14.0%)**	**2 (1.9%)**	**24 (22.4%)**	**107 (100%)**
All PGs									
Normal parenchyma	1	9	–	–	–	–	–	–	10 (10.5%)
Hypoechoic parenchyma	–	7	12	11	2	5	1	–	38 (40.0%)
Heterogeneous parenchyma	–	–	3	8	–	8	1	11	31 (32.6%)
Atrophic parenchyma	–	–	–	1	–	2	–	13	16 (16.8%)
*Total*	**1 (1.1%)**	**16 (16.8%)**	**15 (15.8%)**	**20 (21.1%)**	**2 (2.1%)**	**15 (15.8%)**	**2 (2.1%)**	**24 (25.3%)**	**95 (100%)**
All SMGs									
Normal parenchyma	–	–	–	–	–	–	–	–	–
Hypoechoic parenchyma	1	1	2	3	–	–	–	–	7 (58.3%)
Heterogeneous parenchyma	–	1	1	3	–	–	–	–	5 (41.7%)
Atrophic parenchyma	–	–	–	–	–	–	–	–	–
*Total*	**1**	**2**	**3**	**6**	–	–	–	–	**12 (100%)**
*Last* US examination: maximum US findings									
All glands									
Normal parenchyma	–	8	–	–	–	–	–	–	8 (12.5%)
Hypoechoic parenchyma	–	–	6	4	–	1	–	–	11 (17.2%)
Heterogeneous parenchyma	–	–	2	4	–	6	–	6	18 (28.1%)
Atrophic parenchyma	–	–	–	–	–	–	1	26	27 (42.2%)
*Total*	–	**8 (12.5%)**	**8 (12.5%)**	**8 (12.5%)**	–	**7 (10.9%)**	**1 (1.6%)**	**32 (50.0%)**	**64 (100%)**
All PGs									
Normal parenchyma	–	8	–	–	–	–	–	–	8 (13.8%)
Hypoechoic parenchyma	–	–	5	3	–	1	–	–	9 (15.5%)
Heterogeneous parenchyma	–	–	1	3	–	6	–	6	16 (27.6%)
Atrophic parenchyma	–		–	–		–	–	25	25 (43.1%)
*Total*	–	**8 (13.8%)**	**6 (10.3%)**	**6 (10.3%)**	–	**7 (12.1%)**	–	**31 (53.4%)**	**58 (100%)**
All SMGs									
Normal parenchyma	–	–	–	–	–	–	–	–	–
Hypoechoic parenchyma	–	–	1	1	–	–	–	–	2 (33.3%)
Heterogeneous parenchyma	–	–	1	1	–	–	–	–	2 (33.3%)
Atrophic parenchyma	–	–	–	–	–	–	1	1	2 (33.3%)
*Total*	–	–	**2 (33.3%)**	**2 (33.3%)**	–	–	**1 (16.7%)**	**1 (16.7%)**	**6 (100%)**

PG, parotid gland; SE, sialendoscopy; SMG, submandibular gland; US, ultrasound.

**Table 3:** Cross-tabulation: maximum ultrasound findings and duct dilation at the first and last ultrasound examinations in parotid glands and submandibular glands in 67 patients after radioactive iodine therapy.

Maximum US Findings	Normal Parenchyma	Hypoechoic Parenchyma	Heterogeneous Parenchyma	Atrophic Parenchyma	Total
*First* examination, duct dilation					
All glands	4/10 (40%)	37/45 (82.2%)	24/26 (66.7%)	1/16 (6.3%)	66/107 (61.7%)
PGs	4/10 (40%)	34/38 (89.5%)	22/31 (70.97%)	1/16 (6.3%)	61/95 (64.2%)
SMGs	–	3/7 (42.9%)	2/5 (40.0%)	–	5/12 (41.7%)
*Last* examination, duct dilation					
All glands	1/8 (12.5%)	6/11 (54.5%)	10/18 (55.5%)	4/27 (14.8%)	21/64 (32.8%)
PGs	1/8 (12.5%)	6/9 (66.7%)	10/16 (62.5%)	4/25 (16.0%)	21/58 (36.2%)
SMGs	–	0/2	0/2	0/2	0/6

PG, parotid gland; SE, sialendoscopy; SMG, submandibular gland; US, ultrasound.

**Table 4:** Cross-tabulation of duct dilation in ultrasound and sialendoscopic findings after first and last ultrasound examinations of parotid glands and submandibular glands in 67 patients after radioactive iodine therapy.

SE Finding Ductdilation/ SE Finding Gland (n; %)	Normal	Sialodochitis	Sialodochitis with Tendency to Stenosis	Stenosis (Short)	Stenosis (Segmental)	Stenosis (Long)	Stenosis (Complete, without Obliteration)	Duct Obliteration	Duct Dilation/Finding Total
**First US examination**									
**PGs (n; %)**	0/1	9/16 (56.3%)	12/15 (80.%)	19/20 (95%)	2/2	12/15 (80%)	2/2	5/24 (20.8%)	**61/95 (64.2%)**
**SMGs (n; %)**	1/1	1/2	1/3 (33.3%)	2/6 (33.3%)	–	–	–	–	**5/12 (41.7%)**
**all glands (n; %)**	**1/2**	**10/18 (55.6%)**	**13/18** **(72.2%)**	**21/26 (80.8%)**	**2/2**	**12/15 (80%)**	**2/2**	**5/24 (20.8%)**	**66/107 (61.7%)**
**Last US examination**									
**PGs (n; %)**	–	1/8 (12.5%)	5/6 (83.3%)	5/6 (83.3%)	–	4/7 (57.1%)	–	6/31 (19.4%)	**21/58 (36.2%)**
**SMGs (n; %)**	–	–	0/2	0/2	–		0/1	0/1	**0/6**
**all glands (n; %)**	–	**1/8** **(12.5%)**	**5/8** **(62.5%)**	**5/8** **(62.5%)**	–	**4/7 (57.1%)**	**0/1**	**6/32 (18.8%)**	**21/64 (32.8%)**

PG, parotid gland; SE, sialendoscopy; SMG, submandibular gland; US, ultrasound.

**Table 5:** Details of changes in the findings between the first and last ultrasound and sialendoscopy examinations in the parotid glands and submandibular glands in 64 patients after radioactive iodine therapy.

US Findings	PGs (n, %)	SMGs (n, %)	SE Findings	PGs (n, %)	SMGs (n, %)
Deterioration: change from → to			Deterioration: change from → to		
Hypoechoic → heterogeneous	9	1	SD → SD + tendency to stenosis	3	–
Hypoechoic → atrophic	8	1	SD → short stenosis	1	–
Heterogenous → atrophic	11	1	SD + tendency to stenosis → short stenosis	1	–
			SD + tendency to stenosis → long stenosis	2	–
			SD + tendency to stenosis → complete stenosis	–	1
			Short stenosis → long stenosis	2	–
			SD → duct obliteration	1	–
			SD + tendency to stenosis → duct obliteration	4	–
			Short stenosis → duct obliteration	5	1
			Segmental stenosis → duct obliteration	1	–
			Long stenosis → duct obliteration	8	–
			Complete stenosis → duct obliteration	1	–
	**28 (48.3%)**	**3 (50%)**		**29 (50%)**	**2 (33.3%)**
Unchanged			Unchanged		
Normal	4	–	SD	5	–
Hypoechoic	8	1	SD + tendency to stenosis	–	1
Heterogeneous	7	1	Short stenosis	4	3
Atrophic	6	–	Long stenosis	2	–
			Duct obliteration	11	–
	**25 (43.1%)**	**2 (33.3%)**		**22 (37.9%)**	**4 (66.7%)**
Improved: change from → to			Improved: change from → to		
Hypoechoic → normal	4	–	SD + tendency to stenosis → SD	3	–
Heterogeneous → hypoechoic	1	1	Short stenosis → SD + tendency to stenosis	2	–
			Segmental stenosis → SD + tendency to stenosis	1	–
			Complete stenosis → long stenosis	1	–
	**5 (8.6%)**	**1 (16.7%)**		**7 (12.1%)**	–
***Total***	**58 (100%)**	**6 (100%)**		**58 (100%)**	**6 (100%)**

PG, parotid gland; SD, sialodochitis; SE, sialendoscopy; SMG, submandibular gland; US, ultrasound.

## Data Availability

The data presented in this study are available on request from the corresponding author. The data are not publicly available due to the protection of data privacy.

## Supplementary Materials

The following supporting information can be downloaded at: https://www.mdpi.com/article/10.3390/jcm13030657/s1, Video S1: Sialendoscopy, corresponding to Figure 1A, depicts chronic sialodochitis. While the hilum appears normal, findings are visible in the middle and distal part of the duct system. The duct wall appears pale with a tendency to small amounts of discharge and with no narrowing of the lumen. Video S2: Sialendoscopy, corresponding to Figure 2A, depicts chronic sialodochitis with tendency to stenosis: the duct wall appears pale and markedly thickened with a fibrotic aspect in the proximal and middle part of the duct system, resulting in a narrowing of the lumen, but without significant stenosis. Video S3: Sialendoscopy, corresponding to Figure 3A, depicts subacute sialodochitis in the proximal duct, which developed to a more fibrotic than inflammatory stenosis with clearly visible narrowing of the lumen in the middle part of the duct. Video S4: Sialendoscopy, corresponding to Figure 4A, depicts a chronic sialodochitis with a diffuse fibrotic thickened wall and development to a stenosis in the proximal to middle part of the duct system. Video S5: Sialendoscopy, corresponding to Figure 5A, depicts a complete fibrotic stenosis without any duct lumen visible indicating the area of stenosis. After opening of the fibrotic stenosis an obliteration of the duct system is recognizable characterized by missing of epithelial lining and arachnoid formation of fibrotic tissue within the duct lumen.

## References

[1] Adramerinas M., Andreadis D., Vahtsevanos K., Poulopoulos A., Pazaitou-Panayiotou K. (2021). Sialadenitis as a complication of radioiodine therapy in patients with thyroid cancer: Where do we stand?. Hormones.

[2] Riachy R., Ghazal N., Haidar M.B., Elamine A., Nasrallah M.P. (2020). Early Sialadenitis After Radioactive Iodine Therapy for Differentiated Thyroid Cancer: Prevalence and Predictors. Int. J. Endocrinol..

[3] An Y.-S., Yoon J.-K., Lee S.J., Song H.-S., Yoon S.-H., Jo K.-S. (2013). Symptomatic late-onset sialadenitis after radioiodine therapy in thyroid cancer. Ann. Nucl. Med..

[4] Jo K.S., An Y.-S., Lee S.J., Soh E.-Y., Lee J., Chung Y.-S., Kim D.J., Yoon S.-H., Lee D.H., Yoon J.-K. (2014). Significance of Salivary Gland Radioiodine Retention on Post-ablation 131I Scintigraphy as a Predictor of Salivary Gland Dysfunction in Patients with Differentiated Thyroid Carcinoma. Nucl. Med. Mol. Imaging.

[5] Lee H.N., An J.Y., Lee K.M., Kim E.J., Choi W.S., Kim D.Y. (2015). Salivary gland dysfunction after radioactive iodine (I-131) therapy in patients following total thyroidectomy: Emphasis on radioactive iodine therapy dose. Clin. Imaging.

[6] Lee S.M., Lee J.W., Kim S.Y., Han S.W., Bae W.K. (2013). Prediction of risk for symptomatic sialadenitis by post-therapeutic dual 131I scintigraphy in patients with differentiated thyroid cancer. Ann. Nucl. Med..

[7] Van Nostrand D. (2011). Sialoadenitis secondary to ^131^I therapy for well-differentiated thyroid cancer. Oral Dis..

[8] Hyer S., Kong A., Pratt B., Harmer C. (2007). Salivary gland toxicity after radioiodine therapy for thyroid cancer. Clin. Oncol..

[9] Nakada K., Ishibashi T., Takei T., Hirata K., Shinohara K., Katoh S., Zhao S., Tamaki N., Noguchi Y., Noguchi S. (2005). Does lemon candy decrease salivary gland damage after radioiodine therapy for thyroid cancer?. J. Nucl. Med..

[10] Mandel S.J., Mandel L., Mok Y., Pang Y.H., Teh M., Petersson F., Choi J.-S., An H.-Y., Park I.S., Kim Y.-M. (2003). Radioactive Iodine and the Salivary Glands. Thyroid.

[11] Caglar M., Tuncel M., Alpar R. (2002). Scintigraphic Evaluation of Salivary Gland Dysfunction in Patients with Thyroid Cancer After Radioiodine Treatment. Clin. Nucl. Med..

[12] Solans R., Bosch J.A., Galofré P., Porta F., Roselló J., Selva-O’Callagan A., Vilardell M. (2001). Salivary and lacrimal gland dysfunction (sicca syndrome) after radioiodine therapy. J. Nucl. Med..

[13] Newkirk K.A., Ringel M.D., Wartofsky L., Burman K.D. (2000). The Role of Radioactive Iodine in Salivary Gland Dysfunction. Ear Nose Throat J..

[14] Alexander C., Bader J.B., Schaefer A., Finke C., Kirsch C.M. (1998). Intermediate and long-term side effects of high-dose radioiodine therapy for thyroid carcinoma. J. Nucl. Med..

[15] Stephens L.C., Schultheiss T.E., Price R.E., Ang K.K., Peters L.J. (1991). Radiation apoptosis of serous acinar cells of salivary and lacrimal glands. Cancer.

[16] Allweiss P., Braunstein G.D., Katz A., Waxman A. (1984). Sialadenitis following I-131 therapy for thyroid carcinoma: Concise communication. J. Nucl. Med..

[17] Helman J., Turner R.J., Fox P.C., Baum B.J. (1987). 99mTc-pertechnetate uptake in parotid acinar cells by the Na+/K+/Cl- co-transport system. J. Clin. Investig..

[18] Cavalieri R.R., Agopiantz M., Elhanbali O., Demore B., Cuny T., Demarquet L., Ndiaye C., Barbe F., Brunaud L., Weryha G. (1997). Iodine Metabolism and Thyroid Physiology: Current Concepts. Thyroid.

[19] Jhiang S.M., Cho J.Y., Ryu K.Y., DeYoung B.R., Smanik P.A., McGaughy V.R., Fischer A.H., Mazzaferri E.L. (1998). An immunohistochemical study of Na+/I- symporter in human thyroid tissues and salivary gland tissues. Endocrinology.

[20] De la Vieja A., Dohan O., Levy O., Carrasco N., Andrade B.M., Araujo R.L., Perry R.L.S., Souza E.C.L., Cazarin J.M., Carvalho D.P. (2000). Molecular Analysis of the Sodium/Iodide Symporter: Impact on Thyroid and Extrathyroid Pathophysiology. Physiol. Rev..

[21] Nahlieli O., Nazarian Y. (2006). Sialadenitis following radioiodine therapy—A new diagnostic and treatment modality. Oral Dis..

[22] Kim J.W., Han G.S., Lee S.H., Lee D.Y., Kim Y. (2007). Sialoendoscopic Treatment for Radioiodine Induced Sialadenitis. Laryngoscope.

[23] Brozzi F., Rago T., Bencivelli W., Bianchi F., Santini P., Vitti P., Pinchera A., Ceccarelli C. (2013). Salivary glands ultrasound examination after radioiodine-131 treatment for differentiated thyroid cancer. J. Endocrinol. Investig..

[24] Kim D.W. (2015). Ultrasonographic Features of the Major Salivary Glands after Radioactive Iodine Ablation in Patients with Papillary Thyroid Carcinoma. Ultrasound Med. Biol..

[25] Roh S.S., Kim D.W., Baek H.J. (2016). Association of Xerostomia and Ultrasonographic Features of the Major Salivary Glands After Radioactive Iodine Ablation for Papillary Thyroid Carcinoma. Am. J. Roentgenol..

[26] Horvath E., Skoknic V., Majlis S., Tala H., Silva C., Castillo E., Whittle C., Niedmann J.P., González P. (2020). Radioiodine-Induced Salivary Gland Damage Detected by Ultrasonography in Patients Treated for Papillary Thyroid Cancer: Radioactive Iodine Activity and Risk. Thyroid.

[27] Lima G.A.S., López R.V.M., Ozório G.A., de Freitas R.M.C., Willegaignon J., Sapienza M.T., Chammas M.C., Coura-Filho G.B. (2020). Ultrasonography Echotexture as a surrogate for Sialadenitis secondary to 131I Radioiodine Therapy for differentiated Thyroid Cancer: A review and metaanalysis. Clinics.

[28] Lima G.A.S., López R.V.M., de Freitas R.M.C., Willegaignon J., Sapienza M.T., Chammas M.C., Coura-Filho G.B. (2020). Evaluation of Parotid Salivary Gland Echo Texture by Ultrasound Examinations and Correlation with Whole-Body Scintigraphy After Radioiodine Therapy in Patients with Differentiated Thyroid Carcinoma. J. Ultrasound Med..

[29] Koch M., Fauck V., Sievert M., Mantsopoulos K., Iro H., Mueller S. (2023). Ultrasound Changes in Salivary Glands after Radioactive Iodine Treatmrent in Benign Diseases and Differentiated Cancer of Thyroid Glands in Consideration of Dose and Time Dependency. Eur. J. Ultrasound.

[30] Bomeli S.R., Schaitkin B., Carrau R.L., Walvekar R.R. (2009). Interventional sialendoscopy for treatment of radioiodine-induced sialadenitis. Laryngoscope.

[31] Prendes B.L., Orloff L.A., Eisele D.W. (2012). Therapeutic sialendoscopy for the management of radioiodine sialadenitis. Arch. Otolaryngol.–Head Neck Surg..

[32] De Luca R., Vicidomini A., Trodella M., Tartaro G., Colella G. (2014). Sialoendoscopy: A viable treatment for I131 induced sialoadenitis. Br. J. Oral Maxillofac. Surg..

[33] Bhayani M.K., Acharya V., Kongkiatkamon S., Farah S., Roberts D.B., Sterba J., Chambers M.S., Lai S.Y., Kim J.W., Kim J.M. (2015). Sialendoscopy for Patients with Radioiodine-Induced Sialadenitis and Xerostomia. Thyroid.

[34] Wu C.-B., Xi H., Zhou Q., Zhang L.-M. (2015). Sialendoscopy-Assisted Treatment for Radioiodine-Induced Sialadenitis. J. Oral Maxillofac. Surg..

[35] Kim Y., Choi J., Bin Hong S., Hyun I.Y., Lim J. (2016). Salivary gland function after sialendoscopy for treatment of chronic radioiodine-induced sialadenitis. Head Neck.

[36] Cung T.-D., Lai W., Svider P.F., Hanba C., Samantray J., Folbe A.J., Shkoukani M., Raza S.N. (2017). Sialendoscopy in the Management of Radioiodine Induced Sialadenitis: A Systematic Review. Ann. Otol. Rhinol. Laryngol..

[37] Canzi P., Cacciola S., Capaccio P., Pagella F., Occhini A., Pignataro L., Benazzo M. (2017). Interventional sialendoscopy for radioiodine-induced sialadenitis: Quo vadis?. Acta Otorhinolaryngol. Ital..

[38] Li X., Su J.Z., Zhang Y.Y., Zhang L.Q., Zhang Y.Q., Liu D.G., Yu G.Y. (2020). Inflammation grading and sialoendoscopic treatment of (131)I radioiodine-induced sialadenitis. Beijing Da Xue Xue Bao Yi Xue Ban.

[39] Koch M., Zenk J., Iro H. (2009). Algorithms for Treatment of Salivary Gland Obstructions. Otolaryngol. Clin. N. Am..

[40] Koch M., Iro H. (2017). Extended and treatment-oriented classification of parotid duct stenosis. Laryngoscope.

[41] Koch M., Zenk J., Iro H. (2020). Stenosis and stenosis-like lesions in the submandibular duct: Detailed clinical and sialendoscopy-based analysis and proposal for a classification. Oral Surg. Oral Med. Oral Pathol. Oral Radiol..

[42] Kowalczyk D.M., Jordan J.R., Stringer S.P. (2018). Cost-effectiveness of sialendoscopy versus medical management for radioiodine-induced sialadenitis. Laryngoscope.

